# Health Management Planning for Landscape Fire Smoke Events: A Literature Review and Suggested Human‐Centred Framework for Resource Development

**DOI:** 10.1002/puh2.70428

**Published:** 2026-10-09

**Authors:** Anna M. Gjedrem, Fay H. Johnston, Sharon L. Campbell

**Affiliations:** ^1^ Fire Centre University of Tasmania Hobart Tasmania Australia; ^2^ Healthy Environments and Lives National Research Network University of Canberra Canberra Australian Capital Territory Australia; ^3^ Menzies Institute for Medical Research University of Tasmania Hobart Tasmania Australia; ^4^ Centre for Safe Air NHMRC Centre for Research Excellence Hobart Tasmania Australia

**Keywords:** co‐design, design thinking, disaster health planning, health management, human‐centred, landscape fire smoke

## Abstract

Poor air quality arising from landscape fire smoke (LFS) events presents a substantial health burden to at‐risk individuals and communities. However, there are few dedicated health management resources available for priority populations, despite increasing climate‐related landscape fire risks. We conducted a literature review to analyse health management plans and strategies available for at‐risk individuals in Australia, Canada and the United States. Our review identified four resources, of which only two were dedicated LFS health action plans, revealing a critical gap in preparedness. Given this scarcity, we suggest a human‐centred framework to inform LFS‐specific resource development. We propose a design thinking approach to create tailored LFS health action plans for priority populations and healthcare providers and describe the steps a resource development team would take to apply a human‐centred framework. This approach addresses both practical management challenges and socio‐cultural contexts for implementation.

## Graphical abstract

Landscape fire smoke (LFS) events are increasing with climate change, placing priority populations at greater risk of health harm. A literature review across Australia, Canada and the United States found only four LFS health plans, revealing a critical gap. We propose a design thinking framework to co‐design tailored LFS health action plans.

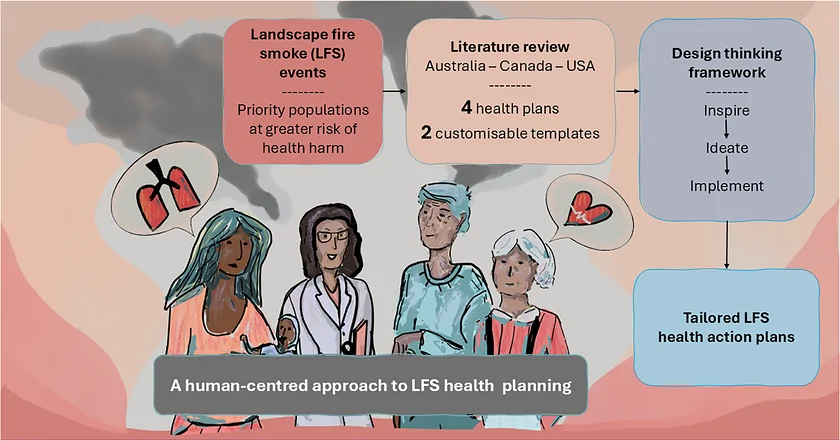

## 1 Introduction

In a warming climate, landscape fire smoke (LFS) events are projected to increase in frequency and severity, a result of increased episodes of unplanned landscape fires [1, 2]. Multiple studies demonstrate that exposure to LFS and other sources of particulate matter (especially particulate matter with a diameter less than 2.5 µm, known as PM_2.5_) can be harmful to the health of exposed populations. For example, Johnston et al. [3] found an estimated 429 deaths as a result of LFS exposure from the 2019 to 20 southeast Australian megafires, resulting in additional healthcare costs of AU$1.95 billion. Health harms are concentrated in particular population groups, including the elderly, young children, those who are pregnant and those living with chronic medical conditions, such as diabetes, lung disease and heart conditions [4]. A number of additional population groups, including those from socioeconomic disadvantage, outdoor workers, people with low literacy, those from cultural and linguistically diverse backgrounds, Indigenous populations and people with disabilities, are also recognised as being at higher risk from poor health outcomes when exposed to LFS, due to increased exposure or difficulty in obtaining and comprehending warning messages [5, 6, 7, 8, 9].

Despite the substantial health burden posed by LFS, health impacts have historically been overlooked in landscape fire adaptation and emergency management discussions. Emergency management has traditionally prioritised the immediate and direct effects of fires, such as loss of life and property near the flame front, while treating smoke primarily as a public health topic rather than an integral component of comprehensive landscape fire management strategies [2]. As global research shifts from mitigation to adaptation in climate change responses, landscape fire management strategies appear to be following this trend, with increasing recognition of the need for multi‐level, intersectoral and evidence‐based interventions [10, 11, 12]. These range from strategic authority‐led initiatives and community‐based support systems to individual behavioural and structural modifications [10]. Governing bodies, such as the Food and Agriculture Organization [12], increasingly incorporate smoke considerations into landscape fire adaptation frameworks, acknowledging that smoke impacts extend beyond individual health concerns to include widespread effects on biodiversity, land degradation, transportation systems and economic activities.

The social and cultural significance of smoke also needs to be considered in adaptation approaches and communication messages [13]. For many Indigenous communities, smoke serves as a visible indicator that land is being properly cared for and maintains both ecological integrity and cultural identity [14, 15]. In Australia, for example, Indigenous cultural burning practices vary significantly across regions, with evidence suggesting that these techniques often produce less smoke than large‐scale prescribed burns conducted by government agencies. A recent study from Tasmania, Australia, found high social acceptance for Indigenous landscape management involving low intensity burns [16]. Successful smoke adaptation requires attention to these social and cultural contexts, with research indicating that ‘matching smoke adaptation resources and strategies to local social contexts can help determine more equitable pathways to living with fire’ [10].

Although health action plans are a common and widely used clinical tool for management of various chronic conditions (e.g., asthma action plans and anaphylaxis action plans [17, 18]), evidence suggests that health‐related planning for LFS events between at‐risk individuals and primary health professionals is either ad hoc or non‐existent, and few readily available guidelines or templates exist to facilitate such planning [19]. On the basis of Canadian evidence [20], an LFS health management plan (also referred to as a bushfire smoke health plan or wildfire smoke action plan), completed with the assistance of a health professional, can promote understanding of LFS preparedness and proactive individual self‐management of symptoms when LFS and other poor air quality events are either forecast or underway. In addition, the plan may protect and improve health during LFS events, potentially alleviating the need for emergency healthcare, and thereby reduce pressure on healthcare services during emergencies. These strategies may also prove valuable for managing other episodes of poor air quality, such as dust storms and wood heater smoke pollution.

The aims of this study are to (1) identify and analyse available resources for managing health risks during LFS events, (2) evaluate gaps in current practice and research and (3) propose a framework for developing tailored, human‐centred health management plans that can support priority populations and healthcare providers in preparing for and responding to LFS events.

## 2 Methods

### 2.1 Literature Search Strategy

We used a literature search to identify available resources for use by people at risk of LFS health impacts and the healthcare professionals who advise these groups. Rather than conducting a comprehensive systematic review, our search aimed to gather sufficient examples to inform innovation in the field and identify gaps in current practice. We conducted targeted searches using both academic and grey literature sources between January and March 2025.

#### 2.1.1 Academic Literature Search

We searched Google Scholar using the following search string: (‘wildfire’ OR ‘bushfire’ OR ‘landscape fire’ OR ‘planned burn’) AND (‘smoke’) AND (‘emergency response plan’ OR ‘health management plan’ OR ‘health plan’) ‐tobacco ‐cigarette. The search was limited to resources from Australia, Canada and the United States, as prior research indicated that most of the available literature regarding LFS health impacts mitigation is concentrated in these countries, and these are the primary English‐speaking countries with substantial landscape fire risk [19]. No date restrictions were applied to enable comprehensive coverage of all relevant resources. A full‐text review was conducted for potentially relevant articles.

#### 2.1.2 Grey Literature Search

To complement the academic literature, we conducted targeted Google searches using similar terms to the academic literature search, engaged in discussions with experts identified through professional networks and employed snowball sampling techniques to identify additional resources.

### 2.2 Screening and Selection Process

#### 2.2.1 Inclusion Criteria

Studies and resources were included if they (1) focused on Australia, Canada or the United States, (2) addressed health effects of LFS exposure, (3) discussed plans or strategies for reducing health risks from LFS and (4) provided guidance or plans suitable for use by at‐risk populations.

The research team discussed and agreed upon these criteria before the search was conducted. A single researcher (AMG) then performed the screening process, with regular consultations with the team to ensure consistency in application of the inclusion criteria.

#### 2.2.2 Exclusion Criteria

Resources were excluded if they primarily focused on specialised plans for workers and firefighters, who are often exposed to harmful chemicals closer to the fire front and therefore require specialised advice; studies without clear relevance to health management planning; and resources focusing solely on smoke exposure without addressing health management strategies. Technical guidance focused solely on the use of face masks and creating cleaner indoor environments also fell outside the scope of this study. Finally, recommendations for fire smoke management during prescribed burning activities were also excluded from this review.

### 2.3 Data Extraction and Analysis

#### 2.3.1 Data Extraction Process

For each included document, information was systematically extracted using the following criteria: (1) if it addressed smoke health effects, (2) if it addressed plans for reducing health risks from LFS and (3) if it suggested specific plans or guidance for implementation.

All resources addressing Criterion 2 (plans for reducing risk) were included in the final review, regardless of whether they also met Criterion 3 (suggesting specific plans). This approach was adopted due to the limited number of resources that provided actual plan templates or detailed guidance. Data were managed using a combination of Word, Excel and EndNote, with structured templates to ensure consistent information extraction across resources.

#### 2.3.2 Analysis Framework

During the analysis, we focused primarily on identifying and categorising existing resources rather than evaluating their quality or effectiveness, as this was beyond the scope of the current research. The synthesis involved thematic organisation of the identified strategies and recommendations, with particular attention to target populations addressed, key recommendations for health protection, format and structure of available plans, implementation considerations and sectoral responsibility (e.g., healthcare and emergency services).

For grey literature, we applied the same criteria used for academic literature, with resources included if they addressed plans for reducing health risks from LFS exposure. Asthma action plans (and other health management plans) were included as analogous resources that could inform the development of LFS health plans but were not themselves primary subjects of the literature search.

## 3 Results

### 3.1 Literature Search—Plans for Reducing Personal Health Risks From LFS Exposure in Australia, Canada and the United States

Our initial Google Scholar search yielded 452 resources, which were screened for 27 relevant documents addressing LFS effects and plans for reducing personal health risks from LFS exposure in Australia, Canada and/or the United States (see Supporting Information, Part A). This body of work shows a growing area of academic interest in the negative health impacts of LFS and the need to incorporate planning and strategies across multiple levels (societal, health services, community and individual user‐groups) to adapt to a changing climate [21, 22, 23, 24, 25, 26]. Although some authors suggested community adaptation pathways to facilitate measures that can be used by and are sensitive to the needs of people at risk [27, 28, 29, 30], others specifically suggested plans or guidelines, such as chronic disease management plans (asthma action plans) [31]; the need to take into account short‐term versus long‐term exposure [32]; the importance of involving healthcare professionals and public health awareness initiatives [28]; and the need for health professionals to triage and prioritise patients during LFS events [33]. However, none of those authors discussed the need for comprehensive recommendations specifically for LFS health plans that may be used by health managers and people at risk of health impacts from LFS exposure.

### 3.2 Current LFS Action Plans in Australia, Canada and the United States

A grey literature search from Australia, Canada and the United States revealed four practical resources (e.g., action plans to prepare for or respond to exposure to LFS) available for people at risk of poor health outcomes through LFS exposure. There are, however, many general recommendations and resources available across different sectors (see Supporting Information, Parts B, C and D for an overview of resources provided by government institutions, healthcare and research institutions in Australia, Canada and the United States).

Of the available resources and plans found in the grey literature review, the content and intent of each plan differed substantially. The first reseource [34] is a commercial web resource providing generic advice for LFS health planning specifically for people with pre‐existing health conditions (asthma or other breathing conditions). In addition, this resource promotes a commercially available product (saline nasal spray), which has a limited evidence base for application in extreme smoke events [35]. However, several of the action recommendations from [34] are similar to other plans [36, 37], including advice to monitor air quality, stay indoors, acquire medications before an adverse event, reduce indoor air pollution, use an air purifier and have P2/N95 masks available. The [34] plan is not designed to be customisable or tailored to specific health needs. The second resource, from the New York State Children's Environmental Health Centres [38], focused on outdoor activities and does not contain many of the recommendations other plans have to reduce adverse health effects (the exception being the advice to monitor local air quality). The third plan, from the Centre for Lung Health [36], provided recommendations for the coexisting impact of heat and smoke exposure. This plan has customisable tick boxes and open text boxes that make it possible for the user to tailor the plan to their specific needs. Although this plan is somewhat text heavy, it is sensitive to health literacy and other vulnerabilities of the user. The fourth plan, from the University of Tasmania [37], is also designed as a customisable resource, however, has less specific information available than the third resource. Although this may increase user‐friendliness, the trade‐off is a reduction in access to important information. A compromise requires sharp design and innovative approaches for a user‐friendly resource, without reducing access to evidence‐based and detailed information. Table 1 contains a summary of the four resources found in the grey literature review.

**TABLE 1:** Landscape fire smoke health action plans in Australia, Canada and the United States.

Country	Sector	Organisation (reference) and link	Title	Format	Approach	Target group	Key recommendations
Australia	Private health industry	[34] https://www.flo.com.au/article/preparing‐for‐bushfire‐season/	Smoke and Health: Preparing for Bushfire Season	Webpage	Bullet point recommendations. Generic approach	People with pre‐existing health conditions such as asthma or other breathing conditions	Create a respiratory health plan ‐ Monitor air quality ‐ Stay indoors if needed ‐ Have medication available ‐ Customise plan to health condition Manage indoor air quality ‐ Keep indoor air clean (close windows; avoid air pollution activities, e.g., burning candles) ‐ Use an air purifier Prepare an emergency kit ‐ P2/N95 masks ‐ Medications ‐ ‐ Salin spray (a nasal spray product marketed by the organisation)
The United States	Healthcare	[38] https://nyscheck.org/rx/	New York State Prescriptions for Prevention—Outdoor air quality action plan	PDF	Bullet point recommendations. Generic approach	People with asthma, lung disease or heart disease	Action plan ‐ Check local air quality report; specific advice to air quality services for people in the United States ‐ Be alert to local wildfires ‐ Plan outdoor activities far away from air pollution sources like the highway ‐ Help reduce air pollution in your neighbourhood (specific advice provided) Resources: Links provided for more information
Canada	Healthcare	[36] https://centreforlunghealth.ca/wildfire‐smoke‐and‐extreme‐heat‐action‐plan/	Wildfire Smoke and Extreme Heat Action Plan	PDF	Tailored plan to needs. Specific plan relevant to the user	Not specified	Know my air quality and temperature ‐ Specific advice to air quality services for Canadians My home and supplies are ready ‐ Extra medication ‐ Extra food ‐ Air cleaner (HEPA filter) ‐ Supply of well‐fitted masks (N95) ‐ Designated cleaner air/cool room ‐ Discuss work‐related measures with employer I have a plan for during an event ‐ Check with and update my buddy ‐ Protective measures if I must go outside: mask; to‐go bag; safe transport options Emergency and non‐emergency contacts provided
Australia	Health Research	[37] https://bushfirehealth.mooc.utas.edu.au/index.php	Bushfire Smoke Health Plan	PDF	Tailored plan to needs. Specific plan relevant to the user	Not specified	Suggested actions undertaken before the bushfire season: ‐ Make an action plan ‐ Seal up air gaps ‐ Consider purchasing an air cleaner ‐ Choose a space in the house best suited as a dedicated clean air space ‐ Identify a public building that has clean air ‐ Get familiar with air quality information Suggested actions undertaken during a smoke event ‐ Track air quality ‐ Reduce smoke exposure Feature: air quality matrix with options according to adverse air quality categoriesStay indoors with closed windows and doors; use air cleaner (different options mentioned); use P2/N95 face mask; seek out clean air environments; consider relocatingFeature open text boxes for medication plan discussed with general practitioner Suggested actions undertaken after the smoke event: ‐ Open up the house for fresh air ‐ Check air quality regularly ‐ Reflect on experiences and update plan (text box available) ‐ Seek help if experiencing worsening physical or mental health impact

Our search found relatively few practical resources for GPs and at‐risk populations, which created the need to include generic respiratory action plans without a direct focus on LFS exposure. Available action plans tailored for at‐risk groups include those from the American Lung Association [39, 40], Asthma Australia [41], Asthma Society of Canada [42], Lung Health Foundation [43], National Asthma Council of Australia [44] and the National Heart, Lung and Blood Institute [45]. Plans adapted to adverse weather events, which can further inform development of LFS health plans, include those from Boston Children's Hospital [46] and the National Collaborating Centre for Environmental Health [47].

We found no landscape fire action plans provided by fire and emergency services that directly addressed LFS health plans directly. This might be partly explained by the fact that LFS health plans are often considered to be relevant for people that are affected by smoke but not directly threatened by the fire event. One notable difference between the United States, Canada and Australia is that online resources for fire emergency personnel in the United States often refer to the hazard posed by LFS in addition to the threat of heat, flame and ember exposure. In Canada and Australia, health effects of smoke exposure planning were predominantly communicated by healthcare organisations. This might reflect a division of responsibility between sectors, and further exploration of this trend is needed for better understanding.

## 4 Discussion

We identified four resources from Australia, Canada and the United States that provided advice to individuals on managing symptoms in the event of LFS exposure. Two of these (one in Australia [37] and one in Canada [36]) are both novel in their approaches and act as a guideline or template to create an LFS health management plan used by priority populations and healthcare providers. The other two resources were designed as generic bullet point lists [34, 38]. This suggests that prior planning for such events is not common practice and is either ad hoc or traditionally non‐existent in high‐risk, English‐speaking countries. However, adaptation actions and strategies to reduce individual and community exposure to LFS are central to successfully navigating a warming future with increased risk of landscape fires, suggesting a critical gap in practice.

### 4.1 Insights From Literature Relevant for LFS Public Health Planning

The literature review justified the need for LFS health plans, demonstrating a body of comprehensive work has been undertaken to identify the adverse effects of LFS health exposure and the alleviating effect of customisable plans for the end‐user. Nevertheless, there remains a paucity of evidence‐based resources that provide a framework for a customisable LFS plan for at‐risk individuals. As the grey literature search revealed, the process of creating such plans is in their early stages. Developing such resources will be the result of collaborative efforts, including insights from end‐users, public health experts, epidemiologists, professional health workers, designers, social researchers and others.

Successful health management during poor air quality episodes, therefore, requires a comprehensive approach, combining individual‐level interventions with broader community‐ and system‐level strategies. This may include developing detailed LFS health plans, implementing targeted communication strategies and integrating digital health technologies for monitoring and communication [19, 32, 48]. Furthermore, healthcare systems must enhance their preparedness through specific protocols and guidelines, with a particular focus on equity in health management planning and implementation [26].

Our synthesis of the current literature underscores the complexity of managing health during poor air quality episodes. Success depends on clear communication, targeted support for vulnerable populations, coordinated healthcare system responses and stakeholder involvement [7, 49, 50]. A recent study by Goodman et al. [19] investigated measures that can reduce health risk during landscape fires, with a focus on global literature on communication and behavioural responses, ventilation and filtration and respiratory protective equipment. The study found that ‘straightforward (non‐technical) public messaging is not only effective in reducing exposure, […] it can reduce self‐reported adverse health effects’, suggesting that such messaging is most effective among people with medical conditions (e.g., asthma). Moreover, Goodman et al. [19] found there is a lack of best practice and efficient communication guidelines, and that most health communications do not adhere to health literacy principles such as readability, accessibility and actionability. There is an additional need to be sensitive concerning advice identifying the user group (e.g., age, socioeconomic status, cultural or linguistic background and location) and the platform from which they might be receiving information (e.g., online or face‐to‐face with a health professional) [7].

As climate change continues to impact air quality globally through longer landscape fire seasons, the importance of well‐designed health management plans becomes increasingly critical for public health protection [2]. At the individual level, several management strategies have shown promise in mitigating health impacts. For example, Forsberg et al. [31] emphasise the importance of implementing existing chronic disease management plans, particularly asthma action plans, which can help prevent exacerbations during poor air quality events. For elderly populations, Madani Hosseini et al. [23] recommend the use of N95/P2 masks and portable air cleaners as essential protective measures. Davis and Phillips [51] highlight the significance of indoor air quality management through proper ventilation and air conditioning settings. Additionally, Izah et al. [28] stress the importance of regular symptom monitoring and early recognition of warning signs.

Community‐level approaches play a vital role in comprehensive air quality management [52]. Cooley et al. [27] describes the development of air quality warning systems as essential infrastructure for public health protection. These systems are most effective when combined with multi‐channel communication strategies, the establishment of clean air shelters and the creation of neighbourhood outreach networks. The literature also emphasises the importance of providing evacuation transportation for vulnerable individuals who may not have access to private transportation during adverse air quality emergencies [53]. Several studies investigate the topic through the lens of a changing climate, which increases vulnerability [21, 22, 23, 24, 25, 27, 29, 52].

Healthcare systems serve as crucial components in managing poor air quality events. Cato [33] advocates for the development of specific triage systems for smoke exposure, whereas Lokmic‐Tomkins et al. [54] highlights the growing importance of digital health technologies, such as air quality monitoring apps, in modern healthcare responses. Healthcare providers play a vital role in providing preventive guidance and education to vulnerable populations, with an increasing focus on implementing targeted interventions for high‐risk groups [55, 56].

Several gaps in current knowledge and practice require attention. The literature reveals a pressing need for more specific guidance on LFS preparedness and response for local health entities [57]. Research on the long‐term health impacts of prolonged smoke exposure remains limited [21], and evidence supporting the effectiveness of various intervention strategies requires further development [19]. Additionally, there is a clear need for more inclusive climate disaster planning that considers the diverse needs of different population groups [29].

### 4.2 Proposed Framework for LFS Health Plans: Design Thinking

Given this critical gap in practice, we propose ‘design thinking’ [58]—a human‐centred approach to public health innovation—as a framework to create LFS health action plans, used by people at risk and their healthcare managers to prepare and respond to adverse smoke events. We propose that a design‐thinking approach will facilitate the integration of multiple critical sectors (e.g., end‐users, public health experts and healthcare professionals) needed to develop an effective LFS plan, while addressing the complexity of the task as outlined above.

Design thinking can be described as ‘research through design’ rather than a pure research methodology, acknowledging the gap between design thinking and traditional public health research [59]. Epidemiology often uses hypotheses and provides clear initial objectives to describe research outputs, whereas design thinking builds on testing, using iterative steps for developing outputs. Design thinking is loose in its application, and the framework does not build upon a strict approach for facilitation. As an example, a recent study published after our literature search was completed [20] found success in combining insights from epidemiology with feedback from user groups to create and implement the Canadian LFS and heat health planning resource identified in our literature search [36].

Design thinking, delivered through qualitative research approaches (such as ethnography, phenomenology, participatory‐observation research and case studies), provides the depth and nuance required to understand local contexts and develop effective interventions [10]. This approach complements the quantitative methods that dominate current research on LFS health impacts and planning, an approach that also dominates in climate change research [60, 61]. Design thinking provides a pragmatic middle ground between research and practice by providing a systematic process for innovation in developing evidence‐based and applied resources. Primarily, design thinking puts humans at the centre of the issues investigated and offers a potential avenue to create new tools and resources by focussing on the needs and contexts of end‐users, while anchoring these in epidemiological evidence [59]. Evidenced‐based and co‐designed strategies are vital to enable at‐risk individuals to reduce their exposure and manage their health during adverse air pollution events [58]. Ideally, these LFS health frameworks are also supplemented by a focus on health literacy [62, 63], in order for the tools and resources to be utilised by those most at risk.

The design thinking framework encompasses existing knowledge and practice to inform an issue, simultaneously integrating user group feedback facilitated through three main phases: inspiration, ideation and implementation. As an example of a design thinking processes, creating tailored LFS health action plans can be facilitated through the following steps (adapted from IDEO U [64]):

1. Frame a question: Set the direction of the design process. For the purpose of developing LFS health plans, we may ask: ‘How may a LFS health plan be best tailored to user needs?’
2. Gather inspiration: Collect information related to the question (e.g., through a literature review, identifying existing resources and programmes and consulting experts). The following questions can inform the discovery of available resources:
  - What is the current state of the evidence?
  - Are there other such plans out there?
  - Are there similar plans out there?
3. Generate ideas: Interview and consolidate the target user groups of the plan (e.g., people at risk of adverse health effects from LFS; primary health providers that can use these plans in their consultations). The following questions can inform the understanding valuable content and platforms for integrating LFS health plans:
  - What do consumer groups need?
  - What do healthcare providers need?
  - How can this be integrated?
4. Make ideas tangible: Create a prototype of the resource.
5. Test and learn: Show and gain feedback on the ‘pilot plan’ from user groups, experts and health providers. Develop the resource iteratively.
6. Share the story: Making sure the resources are available and known by the people who can benefit from it. Examples of this for the LFS health plan include:
  - Sharing the resource to health professionals and people at risk across platforms
  - Creating or getting involved in a community of experts/practice to discuss and learn from each other
  - Sharing the plans through campaigns/platforms in the healthcare sector and make plans available for the broader public through peak consumer bodies or health websites.

### 4.3 Policy Implication and Recommendations

As the global climate continues to change, proactive planning for LFS events becomes increasingly critical for public health protection. By developing accessible and actionable health management plans, there is high potential to better equip communities and healthcare systems to mitigate the adverse health impacts of LFS and other air quality emergencies.

The synthesis of available literature demonstrates that effective health management during poor air quality episodes requires a multi‐layered approach. Individual‐level interventions, such as advancing LFS health plans, must be complemented by broader system‐level strategies, including coordinated healthcare responses and community support networks. With coordinated effort across sectors and disciplines, we can develop more robust approaches to protecting public health in the face of increasing environmental challenges. Particular attention must be paid to vulnerable groups, including individuals with pre‐existing chronic health conditions, the elderly, children, those who are pregnant, those from socioeconomically disadvantaged populations, outdoor workers, people with low literacy, those from cultural and linguistically diverse backgrounds, Indigenous communities and people with disabilities.

On the basis of our findings, we recommend:

1. Local development of standardised ‘LFS health action plans’ that can be easily customised for individual needs
2. Increased collaboration between healthcare systems, emergency services and community organisations to ensure coordinated integration and response to LFS events
3. Integration of health literacy in all LFS health communication materials to enhance understanding and adoption
4. Prioritisation of inclusive planning approaches that consider the diverse needs of vulnerable populations
5. Investment in research to evaluate the effectiveness of various intervention strategies during poor air quality events

### 4.4 Limitations and Strengths of the Study

It is important to note that this study was not designed as a comprehensive systematic review but rather as an exploratory investigation to inform innovation in the field of LFS health planning. By combining academic and grey literature searches, we captured both scholarly perspectives and practical resources currently available to the public and healthcare providers. Our focus on both individual‐level interventions and system‐level responses acknowledges the multifaceted nature of effective smoke management strategies. The methodology was purposefully targeted to identify examples of current practice that could spark innovation and highlight gaps requiring attention.

The focus on three English‐speaking countries (Australia, Canada and the United States) was intentional, as these regions have documented experience with LFS events and related health planning efforts. Although this limits generalisability to other regions, it provides a focused examination of contexts where such planning is actively being developed.

As the primary aim was to inform innovation rather than evaluate effectiveness, the study did not formally assess the quality or effectiveness of identified resources. This represents an important area for future research to understand the contextual factors that influence the successful adoption of LFS health plans across different populations and settings.

This study provides a valuable foundation for understanding the current landscape of LFS health management plans and offers evidence‐based recommendations for improving preparedness and response strategies for vulnerable populations. By suggesting ‘design thinking’ we provide concrete, actionable guidance for developing user‐centred health management plans that are more likely to be implemented effectively. Building on this study's suggested approach and insights a LFS health managment plan was created [65].

## 5 Conclusion

An LFS health plan provides a practical approach to increasing the health and wellbeing of individuals and reduces healthcare treatment during significant LFS events. Our literature search identified few examples of LFS health plans, highlighting the critical need for an evidence‐based approach to developing such resources. Design thinking is a recommended pathway to the development of new planning tools to prepare at‐risk individuals for LFS events.

## Author Contributions

**Anna M. Gjedrem**: investigation, writing – original draft, methodology, writing – review and editing, funding acquisition, formal analysis, validation, data curation. **Fay H. Johnston**: conceptualisation, funding acquisition, supervision, writing – review and editing. **Sharon L. Campbell**: conceptualisation, funding acquisition, supervision, validation, writing – review and editing, project administration, methodology.

## Funding

Open access publishing facilitated by University of Tasmania, as part of the Wiley ‐ University of Tasmania agreement via the Council of Australasian University Librarians

We acknowledge funding support from the Centre for Safe Air, NHMRC Centre for Research Excellence. We also acknowledge support from the HEAL (Healthy Environments and Lives) National Research Network, which receives funding from the National Health and Medical Research Council (NHMRC) Special Initiative in Human Health and Environmental Change (2008937).

## Ethics Statement

The authors have nothing to report.

## Consent

The authors have nothing to report.

## Conflicts of Interest

The authors declare no conflicts of interest.

## Supporting information




**Supporting File:** puh270428‐sup‐0001‐SuppMat.docx.

## Data Availability

Data sharing is not applicable to this article as no datasets were generated or analysed during the current study.
