# Supplementary material for: Health Management Planning for Landscape Fire Smoke Events: A Literature Review and Suggested Human‐Centred Framework for Resource Development
Source: Public Health Chall. 2026 Oct 9;5(4):e70428. doi: 10.1002/puh2.70428 (PMC13652956; doi:10.1002/puh2.70428)
Supplement: Supplementary file 1 [file PUH2-5-e70428-s001.docx]

**Supplementary Materials**

**Health management planning for landscape fire smoke events: A literature review and suggested human-centred framework for resource development**

Anna M Gjedrem^1,4^, Fay H Johnston^1,2,3,4^, Sharon L Campbell^1,2,3,4^

^1^Fire Centre, University of Tasmania, Hobart, Tasmania, Australia

^2^Menzies Institute for Medical Research, University of Tasmania, Hobart, Tasmania, Australia

^3^Centre for Safe Air, NHMRC Centre for Research Excellence, Hobart, Tasmania, Australia

^4^Healthy Environments and Lives National Research Network, University of Canberra, Canberra, ACT, Australia

**Corresponding author**

Dr Anna M Gjedrem

Fire Centre, Discipline of Biology, School of Natural Sciences, College of Science and Engineering, University of Tasmania, Private Bag 55, Hobart, Tas 7001, Australia.

Email: [anna.gjedrem@utas.edu.au](mailto:anna.gjedrem@utas.edu.au)

**Supplementary Materials**

**Health management planning for landscape fire smoke events: A literature review and suggested human-centred framework for resource development**

Anna M Gjedrem^1,4^, Fay H Johnston^1,2,3,4^, Sharon L Campbell^1,2,3,4^

^1^Fire Centre, University of Tasmania, Hobart, Tasmania, Australia

^2^Menzies Institute for Medical Research, University of Tasmania, Hobart, Tasmania, Australia

^3^Centre for Safe Air, NHMRC Centre for Research Excellence, Hobart, Tasmania, Australia

^4^Healthy Environments and Lives National Research Network, University of Canberra, Canberra, ACT, Australia

A. **Academic literature search results**

| **Number** | **Source** | **Title** | **1.Addresses smoke health effects** | **2.Addresses plans for reducing risk to health from LFS** | **3.Suggest plan or guidance** |
| --- | --- | --- | --- | --- | --- |
| 1 | (Branch, 2008) | Public Health Climate Change Adaptation Strategy for California | Yes | Yes |  |
| 2 | (Burns, 2022) | The role of general practitioners in disaster health management | Yes | Yes |  |
| 3 | (Cato, 2002) | Examination of the" all hazards" approach to disaster management as applied to field disaster management and pre-hospital care in Australia | Yes | Yes | Yes |
| 4 | (Cooley et al., 2012) | Community-Based Climate Adaptation Planning: Case Study of Oakland, California | Yes | Yes |  |
| 5 | (Davis & Phillips, 2009) | Effective Emergency Management: Making Improvements for Communities and People with Disabilities | Yes | Yes |  |
| 6 | (Dhingra et al., 2023) | Wildfire smoke exposure and early childhood respiratory health: a study of prescription claims data | Yes | Yes |  |
| 7 | (Dirmyer, 2019) | Asthma vs. PM2.5: A Bridge Between Health and Environmental Surveillance | Yes | Yes |  |
| 8 | (Feo et al., 2023) | The Human Health Benefits of Improving Forest Health in California: Investigating the Links Between Forest Management, Wildfire Smoke, and the Health Sector | Yes | Yes |  |
| 9 | (Forsberg et al., 2012) | Wildfire smoke exposure: A guide for the nurse practitioner | Yes | Yes | Yes |
| 10 | (Goh et al., 1999) | Health Guidelines | Yes | Yes | Yes |
| 11 | (Heaney et al., 2021) | Efficacy of communication techniques and health outcomes of bushfire smoke exposure: a scoping review | Yes | Yes |  |
| 12 | (Holmes et al., 2022) | Progress of Local Health Department Planning Actions for Climate Change: Perspectives from California, USA | Yes | Yes |  |
| 13 | (Hutchinson et al., 2018) | The San Diego 2007 wildfires and Medi-Cal emergency department presentations, inpatient hospitalizations, and outpatient visits: An observational study of smoke exposure periods and a bidirectional case-crossover analysis | Yes | Yes |  |
| 14 | (Izah et al., 2024) | Short-term health effects of air pollution | Yes | Yes | Yes |
| 15 | (Jiao et al., 2024) | Fine Particulate Matter, Its Constituents, and Spontaneous Preterm Birth | Yes | Yes |  |
| 16 | (Lee et al., 2023) | Impacts of climate change on health and health services in northern New South Wales, Australia: a rapid review | Yes | Yes |  |
| 17 | (Levi et al., 2009) | Health Problems Heat Up | Yes | Yes |  |
| 18 | (Lokmic-Tomkins et al., 2023) | Lessons learned from natural disasters around digital health technologies and delivering quality healthcare | Yes | Yes |  |
| 19 | (Madani Hosseini et al., 2024) | Climate crisis risks to elderly health: strategies for effective promotion and response | Yes | Yes |  |
| 20 | (Mahmood et al., 2023) | Unravelling the link: exploring the effects of environmental change on the cardiovascular system | Yes | Yes |  |
| 21 | (McBean et al., 2021) | Building Climate Resilient Communities: Living Within the Earth’s Carrying Capacity | Yes | Yes |  |
| 22 | (McManus, 2023) | Science and Science Communication of Anthropogenic Climate Change and Extreme Weather-Related Events: A Critical Discourse Analysis of the Alpha Road/Tambaroora Bushfire in Australia, 2023 | Yes | Yes |  |
| 23 | (Stares et al., 2014) | Evidence Review: Use of evacuation to protect public health during wildfire smoke events. | Yes | Yes |  |
| 24 | (Summer, 2020) | Connecting with Life: Finding Nature in an Urban World | Yes | Yes |  |
| 25 | (Weeramanthri et al., 2020) | Climate health WA inquiry | Yes | Yes |  |
| 26 | (Wendel, 2022) | How Will You Know Whether Your Efforts to Strengthen Patient Health Are Effective and Efficient? How Will You Convince Others? | Yes | Yes |  |
| 27 | (Zuber, 2018) | Protecting American Health from Climate Change: What is needed to expand adaptation planning by US state and local health departments? | Yes | Yes |  |

**References**

Branch, D. W. (2008). Public Health Climate Change Adaptation Strategy for California. *Center for Chronic Disease Prevention and Health Promotion*.

Burns, P. L. (2022). *The role of general practitioners in disaster health management* The Australian National University (Australia)].

Cato, D. (2002). *Examination of the" all hazards" approach to disaster management as applied to field disaster management and pre-hospital care in Australia* Deakin University].

Cooley, H., Garzón, C., Heberger, M., Allen, L., & Doty, A. (2012). Community-Based Climate Adaptation Planning: Case Study of Oakland, California.

Davis, E., & Phillips, B. (2009). Effective Emergency Management: Making Improvements for Communities and People with Disabilities. *National Council on Disability*.

Dhingra, R., Keeler, C., Staley, B. S., Jardel, H. V., Ward-Caviness, C., Rebuli, M. E., Xi, Y., Rappazzo, K., Hernandez, M., & Chelminski, A. N. (2023). Wildfire smoke exposure and early childhood respiratory health: a study of prescription claims data. *Environmental Health*, *22*(1), 48.

Dirmyer, V. F. (2019). Asthma vs. PM2. 5: A Bridge Between Health and Environmental Surveillance. *Online Journal of Public Health Informatics*, *11*(1), e62471.

Feo, T. J., Quesnel Seipp, K., Stroming, S., Montgomery, J., Graff Zivin, J., Huber-Stearns, H., Kochanski, A., Tompkins, R., Wu, J., & Mace, A. J. (2023). *The Human Health Benefits of Improving Forest Health in California: Investigating the Links Between Forest Management, Wildfire Smoke, and the Health Sector*.

Forsberg, N. T., Longo, B. M., Baxter, K., & Boutté, M. (2012). Wildfire smoke exposure: A guide for the nurse practitioner. *The Journal for Nurse Practitioners*, *8*(2), 98-106.

Goh, K.-T., Schwela, D., Goldammer, J. G., & Simpson, O. (1999). HEALTH GUIDELINES.

Heaney, E., Hunter, L., Clulow, A., Bowles, D., & Vardoulakis, S. (2021). Efficacy of communication techniques and health outcomes of bushfire smoke exposure: a scoping review. *International Journal of Environmental Research and Public Health*, *18*(20), 10889.

Holmes, T. J., Holt, A., & English, D. Q. (2022). Progress of Local Health Department Planning Actions for Climate Change: Perspectives from California, USA. *International Journal of Environmental Research and Public Health*, *19*(13), 7984.

Hutchinson, J. A., Vargo, J., Milet, M., French, N. H., Billmire, M., Johnson, J., & Hoshiko, S. (2018). The San Diego 2007 wildfires and Medi-Cal emergency department presentations, inpatient hospitalizations, and outpatient visits: An observational study of smoke exposure periods and a bidirectional case-crossover analysis. *PLoS medicine*, *15*(7), e1002601.

Izah, S. C., Ogwu, M. C., Etim, N. G., Shahsavani, A., & Namvar, Z. (2024). Short-term health effects of air pollution. In *Air Pollutants in the Context of One Health: Fundamentals, Sources, and Impacts* (pp. 249-278). Springer.

Jiao, A., Reilly, A. N., Benmarhnia, T., Sun, Y., Avila, C., Chiu, V., Slezak, J., Sacks, D. A., Molitor, J., & Li, M. (2024). Fine Particulate Matter, Its Constituents, and Spontaneous Preterm Birth. *JAMA Network Open*, *7*(11), e2444593-e2444593.

Lee, G. W., Vine, K., Atkinson, A.-R., Tong, M., Longman, J., Barratt, A., Bailie, R., Vardoulakis, S., Matthews, V., & Rahman, K. M. (2023). Impacts of climate change on health and health services in northern New South Wales, Australia: a rapid review. *International Journal of Environmental Research and Public Health*, *20*(13), 6285.

Levi et al. (2009). *Health Problems Heat Up*.

Lokmic-Tomkins, Z., Bhandari, D., Bain, C., Borda, A., Kariotis, T. C., & Reser, D. (2023). Lessons learned from natural disasters around digital health technologies and delivering quality healthcare. *International Journal of Environmental Research and Public Health*, *20*(5), 4542.

Madani Hosseini, M., Zargoush, M., & Ghazalbash, S. (2024). Climate crisis risks to elderly health: strategies for effective promotion and response. *Health Promotion International*, *39*(2), daae031.

Mahmood, R., Said, A., Kanagala, S. G., Gupta, V., & Jain, R. (2023). Unravelling the link: exploring the effects of environmental change on the cardiovascular system. *Future cardiology*, *19*(13), 649-659.

McBean, G., Kovacs, P., Voogt, J., Kopp, G., & Guilbault, S. (2021). Building Climate Resilient Communities: Living Within the Earth’s Carrying Capacity.

McManus, P. (2023). Science and Science Communication of Anthropogenic Climate Change and Extreme Weather-Related Events: A Critical Discourse Analysis of the Alpha Road/Tambaroora Bushfire in Australia, 2023. *Tambaroora Bushfire in Australia*.

Stares, J., Erland, R., Mazey, P., & Rideout, K. (2014). Evidence Review: Use of evacuation to protect public health during wildfire smoke events. *Vancouver BC: BC Centre for Disease Control*.

Summer, M. (2020). *Connecting with Life: Finding Nature in an Urban World*. Summer Press.

Weeramanthri, T., Joyce, S., Bowman, F., Bangor-Jones, R., & Law, C. (2020). *Climate health WA inquiry*. Department of Health, Government of Western Australia Perth, WA, Australia.

Wendel, J. (2022). How Will You Know Whether Your Efforts to Strengthen Patient Health Are Effective and Efficient? How Will You Convince Others? In *Clinical Health Psychology in Military and Veteran Settings: Innovations for the Future* (pp. 115-144). Springer.

Zuber, A. (2018). *Protecting American Health from Climate Change: What is needed to expand adaptation planning by US state and local health departments?* The University of North Carolina at Chapel Hill].

**Supplementary Materials**

**Health management planning for landscape fire smoke events: A literature review and suggested human-centred framework for resource development**

Anna M Gjedrem^1,4^, Fay H Johnston^1,2,3,4^, Sharon L Campbell^1,2,3,4^

^1^Fire Centre, University of Tasmania, Hobart, Tasmania, Australia

^2^Menzies Institute for Medical Research, University of Tasmania, Hobart, Tasmania, Australia

^3^Centre for Safe Air, NHMRC Centre for Research Excellence, Hobart, Tasmania, Australia

^4^Healthy Environments and Lives National Research Network, University of Canberra, Canberra, ACT, Australia

B. **Resource library – LFS and health impacts in Australia**

**Healthcare institutions**

| **Stakeholder** | **General information and Warning systems** | **Resources (e.g. fact sheets/ guidance)** | **LFS action plans** | **Other action plans** |
| --- | --- | --- | --- | --- |
| Hearth foundation | **Environment, climate change and heart health:** https://www.heartfoundation.org.au/healthy-living/physical-activity/climate-change-and-heart-health | **Bushfire smoke and your health:** https://www.alpinehealth.org.au/Portals/0/Alpine-Health/Documents/Media%20Release/Bushfire_smoke_and_your_health_fact_sheet_HF.PDF |  |  |
| Lung Foundation Australia | **How to protect your lung health this bushfire season:** https://lungfoundation.com.au/blog/how-to-protect-your-lung-health-this-bushfire-season/ | **Check in with your lungs**: https://lungfoundation.com.au/wp-content/uploads/2018/10/Flyer-Lung-Health-Checklist-Nov2018.pdfhttps://lungfoundation.com.au/wp-content/uploads/2018/10/Flyer-Lung-Health-Checklist-Nov2018.pdf |  |  |
| Health direct | **Bushfires and your health:** https://www.healthdirect.gov.au/bushfires-and-your-health |  |  |  |
| National Asthma Council Australia |  | **Bushfires and asthma**: https://www.nationalasthma.org.au/living-with-asthma/resources/patients-carers/factsheets/bushfires-and-asthma  https://www.nationalasthma.org.au/living-with-asthma/resources/patients-carers/factsheets/bushfires-and-asthma |  | **Asthma action plans:** https://www.nationalasthma.org.au/health-professionals/asthma-action-plans/asthma-action-plan-library |
| Asthma Australia |  | **Five ways to get asthma ready for bushfire season**: https://asthma.org.au/blog/five-ways-to-get-asthma-ready-for-bushfire-season/  https://asthma.org.au/blog/five-ways-to-get-asthma-ready-for-bushfire-season/ |  | **Asthma action plan:** https://asthma.org.au/manage-asthma/asthma-action-plan/ |
| Pharmaceutical Resources |  | **Bushfires and asthma:** https://www.sensitivechoice.com/resource/bushfires-and-asthma/ | **Smoke and Health: Preparing for Bushfire Season** https://www.flo.com.au/article/preparing-for-bushfire-season/ |  |

**Research institutions**

| **Stakeholder** | **General information and warning systems** | **Resources (e.g. fact sheets/ guidance)** | **LFS action plans** | **Other action plans** |
| --- | --- | --- | --- | --- |
| Centre for Safe Air |  | **How to cope with bushfire smoke:** https://safeair.org.au/how-to-cope-with-bushfire-smoke/  **Bushfire smoke: know your health risk and make a plan:** https://safeair.org.au/bushfire-smoke-know-your-health-risk-and-make-a-plan/ |  |  |
| Australian National University: National Centre for Epidemiology and Population Health. |  | **How to protect yourself and others from bushfire smoke:** https://nceph.anu.edu.au/news-events/news/how-protect-yourself-and-others-bushfire-smokehttps://nceph.anu.edu.au/news-events/news/how-protect-yourself-and-others-bushfire-smoke  **What is bushfire smoke and how harmful is it:**  https://nceph.anu.edu.au/files/2020_Bushfire_Smoke_Fact.pdf  **How to monitor local conditions:** https://nceph.anu.edu.au/files/2020_Bushfire_Smoke_Fact_monitoring.pdf |  |  |
| University of Tasmania: Menzies Institute for Medical Research |  | **Bushfires and your health**: https://bushfirehealth.mooc.utas.edu.au/index.phphttps://bushfirehealth.mooc.utas.edu.au/index.php | **Bushfire Smoke Health Plan:** https://bushfirehealth.mooc.utas.edu.au/index.php |  |

**Government authorities**

| **Stakeholder** | **General information and warning systems** | **Resources (e.g. fact sheets/ guidance)** | **LFS smoke action plans** | **Other action plans** |
| --- | --- | --- | --- | --- |
| Department of Health and Aged Care | **Air quality:** https://www.health.gov.au/topics/environmental-health/about/air-quality https://www.health.gov.au/topics/environmental-health/about/air-quality | **enHealth guidance – Guidance for public health agencies – Managing prolonged smoke events from landscape fires:**  https://www.health.gov.au/resources/publications/enhealth-guidance-guidance-for-public-health-agencies-managing-prolonged-smoke-events-from-landscape-fires?language=en  **Guidance for pregnant women and young children:** https://www.pregnancybirthbaby.org.au/pregnant-women-children-and-bushfires |  |  |
| Bureau of Meteorology | **Smoke and air quality information:** http://www.bom.gov.au/catalogue/warnings/air-pollution.shtml |  |  |  |
| AirRater | **Warnings**: https://airrater.org/ |  |  |  |
| NSW Department of Health | **Protect yourself from bushfire smoke:** https://www.health.nsw.gov.au/environment/bushfire/Pages/bushfire-protection.aspx https://www.health.nsw.gov.au/environment/bushfire/Pages/bushfire-protection.aspx  **Health advice:** https://www.airquality.nsw.gov.au/health-advice |  |  |  |
| NT Department of Health | **Staying safe from bushfire smoke:** https://nt.gov.au/emergency/bushfire/prepare-and-plan/staying-safe-from-bushfire-smoke |  |  |  |
| QLD Department of Health | **Bushfire smoke and your health:** <https://www.qld.gov.au/health/staying-healthy/environmental/after-a-disaster/bushfires/bushfire-smoke-and-your-health>  **Smoke and dust health action levels explained:** <https://apps.des.qld.gov.au/air-quality/health/about/>  **Air quality:** https://www.health.qld.gov.au/public-health/disaster/public-health-advice/air-quality | **Fact sheet: Staying safe and healthy in smoky bushfire conditions** https://www.health.qld.gov.au/__data/assets/pdf_file/0034/1288726/bushfire-conditions-plain-english.pdf |  |  |
| QLD Government | **Bushfires and dust storms:** https://www.qld.gov.au/environment/management/monitoring/air/air-pollution/bushfires |  |  |  |
| SA Environmental Protection Authority | **Air quality:** https://www.epa.sa.gov.au/environmental_info/air_quality |  |  |  |
| SA Department of Health | **Bushfire smoke and your health:** https://www.sahealth.sa.gov.au/wps/wcm/connect/public+content/sa+health+internet/public+health/bushfires/bushfire+smoke+and+your+healthhttps://www.sahealth.sa.gov.au/wps/wcm/connect/public+content/sa+health+internet/public+health/bushfires/bushfire+smoke+and+your+health |  |  |  |
| TAS Environmental Protection Authority | **Real-time Air Quality Data for Tasmania:** https://epa.tas.gov.au/environment/air/monitoring-air-pollution/monitoring-data/real-time-air-quality-data-for-tasmania https://epa.tas.gov.au/environment/air/monitoring-air-pollution/monitoring-data/real-time-air-quality-data-for-tasmania  **All Air Monitoring Stations - Current Data Table:** https://epa.tas.gov.au/environment/air/monitoring-air-pollution/monitoring-data/real-time-air-quality-data-for-tasmania/all-sites-table |  |  |  |
| TAS Department of Health | **Air quality:** https://www.health.tas.gov.au/health-topics/environmental-health/air-quality#bushfire-smoke-and-your-health | **Bushfire smoke and your health:** https://www.health.tas.gov.au/sites/default/files/2024-11/factsheet_-_bushfire_smoke_and_your_health_-_update_november_2024_-_accessible_0.pdf |  |  |
| Tasmanian Fire Service |  | **Smoke and Asthma:** https://www.fire.tas.gov.au/Show?pageId=colAsthma |  |  |
| VIC Environmental Protection Authority | **Outdoor air and your health:** <https://www.epa.vic.gov.au/for-community/environmental-information/environmental-public-health/air> |  |  |  |
| VIC Department of Health | **Smoke and your health**: https://www.betterhealth.vic.gov.au/bushfiresmoke |  |  |  |
| VIC Forest fire management | **Smoke – How to deal with smoke:** https://www.ffm.vic.gov.au/bushfire-fuel-and-risk-management/smoke | **Protect your health from smoke** https://www.ffm.vic.gov.au/__data/assets/pdf_file/0026/27665/PB-health-flyer-DL_state_2017V6_Web.pdf |  |  |
| WA Environmental Protection Authority | **Factor guidelines and technical guidance: Air** https://www.epa.wa.gov.au/policies-guidance/air |  |  |  |
| WA Department of Health | **Environmental health risks during and after a bushfire:** https://www.health.wa.gov.au/Articles/A_E/Environmental-health-risks-during-and-after-a-bushfire https://www.health.wa.gov.au/Articles/A_E/Environmental-health-risks-during-and-after-a-bushfire  **Smoke hazards from bushfires:** https://www.healthywa.wa.gov.au/Articles/S_T/Smoke-hazard-from-bushfireshttps://www.healthywa.wa.gov.au/Articles/S_T/Smoke-hazard-from-bushfires | **Stay safe from bushfire smoke**: https://www.health.wa.gov.au/~/media/Corp/Documents/Health-for/Environmental-health/Chemical-Hazards/PDF/Bushfire-A4-Poster.pdf |  |  |

**Supplementary Materials**

**Health management planning for landscape fire smoke events: A literature review and suggested human-centred framework for resource development**

Anna M Gjedrem^1,4^, Fay H Johnston^1,2,3,4^, Sharon L Campbell^1,2,3,4^

^1^Fire Centre, University of Tasmania, Hobart, Tasmania, Australia

^2^Menzies Institute for Medical Research, University of Tasmania, Hobart, Tasmania, Australia

^3^Centre for Safe Air, NHMRC Centre for Research Excellence, Hobart, Tasmania, Australia

^4^Healthy Environments and Lives National Research Network, University of Canberra, Canberra, ACT, Australia

C. **Resource library – LFS and health impacts in Canada**

**Healthcare institutions**

| **Stakeholder** | **General information and Warning systems** | **Resources (e.g. fact sheets/ guidance)** | **LFS action plans** | **Other action plans** |
| --- | --- | --- | --- | --- |
| **Centre for Lung Health** |  |  | **Wildfire Smoke and Extreme Heat Action Plan:** <https://centreforlunghealth.ca/wildfire-smoke-and-extreme-heat-action-plan/> |  |
| **Interior Health Canada** |  | **Medical health officers' letter to community leaders on heat, smoke and health** <https://www.interiorhealth.ca/media/medical-health-officers-letter-community-leaders-heat-smoke-and-health> |  |  |
| **Lung Health Foundation** | **Protect your lung health from wildfire smoke and pollution:** <https://hcp.lunghealth.ca/protect-your-lung-health-from-wildfire-smoke-and-pollution/> |  |  | **Lung Health Foundation Child Asthma Action Plan** <https://operationfresh.wpenginepowered.com/wp-content/uploads/2023/02/Pediatric-Asthma-Plan-November-2022.pdf>  **Lung Health Foundation Adult Asthma Action Plan** <https://operationfresh.wpenginepowered.com/wp-content/uploads/2022/03/2022-March31-Adult-Asthma-Action-Plan.pdf> |
| **The BC Medical Journal** |  | **Forest fires: A clinician primer** <https://bcmj.org/bccdc/forest-fires-clinician-primer> |  |  |
| **Northern Inter-Tribal Health Authority** |  | **Wildfire smoke** <https://www.nitha.com/wp-content/uploads/2023/06/Wildfire-Smoke-Safety-Poster.pdf> |  |  |
| **Asthma Society of Canada** |  |  |  | **Asthma Action Plans:** <https://asthma.ca/get-help/living-with-asthma/asthma-action-plan/>  **Kids:** <https://asthma.ca/wp-content/uploads/2020/08/Kids-Action-Plan-FINAL-2022.pdf>  **Adults:** <https://asthma.ca/wp-content/uploads/2020/06/Asthma-Action-Plan_optimized.pdf> |

**Research institutions**

| **Stakeholder** | **General information and warning systems** | **Resources (e.g. fact sheets/ guidance)** | **LFS action plans** | **Other action plans** |
| --- | --- | --- | --- | --- |
| **The University of British Columbia** | **Wildfire Smoke Health & Safety**  <https://srs.ubc.ca/health-safety/occupational-hygiene/wildfire-smoke-health-safety/#:~:text=Once%20the%20risk%20of%20wildfire%20smoke%20has%20been,to%20ensure%20the%20risk%20is%20eliminated%20or%20mitigated>. |  |  |  |

**Government authorities**

| **Stakeholder** | **General information and Warning systems** | **Resources (e.g. fact sheets/ guidance)** | **LFS action plans** | **Other action plans** |
| --- | --- | --- | --- | --- |
| **The Government of Canada** | **Wildfire smoke, air quality and your health: Overview** <https://www.canada.ca/en/services/health/healthy-living/environment/air-quality/wildfire-smoke.html>  **Wildfire smoke with extreme heat:** <https://www.canada.ca/en/health-canada/services/publications/healthy-living/combine-wildfire-smoke-heat.html>  **Wildfire smoke, air quality and your health: Health effects of wildfire smoke exposure** <https://www.canada.ca/en/services/health/healthy-living/environment/air-quality/wildfire-smoke/health-effects-exposure.html>  **Climate Change, Wildfires and Canadians’ Health**  <https://changingclimate.ca/site/assets/uploads/sites/5/2022/02/Wildfires-Infographic-Fact-Sheet-EN.pdf>  **Air Quality Health Index** <https://www.canada.ca/en/environment-climate-change/services/air-quality-health-index.html>  **Air Quality Model Forecast Maps** <https://weather.gc.ca/firework/index_e.html> | **Get prepared** <https://www.getprepared.gc.ca/cnt/hzd/wldfrs-prp-en.aspx>  **Wildfire smoke, air quality and your health: Preparing for wildfire smoke events:** <https://www.canada.ca/en/services/health/healthy-living/environment/air-quality/wildfire-smoke/preparing-events.html>  **Wildfire smoke, air quality and your health:** <https://www.canada.ca/en/services/health/healthy-living/environment/air-quality/wildfire-smoke/protecting-your-physical-mental-health.html>  **Wildfires in Canada: Toolkit for Public Health Authorities**  <https://www.canada.ca/en/public-health/services/publications/healthy-living/wildfires-canada-toolkit-public-health-authorities.html>  **Wildfire smoke with extreme heat** <https://www.canada.ca/content/dam/hc-sc/documents/services/publications/healthy-living/combine-wildfire-smoke-heat/combine-wildfire-smoke-heat.pdf> |  |  |
| **National Collaborating Centre for Environmental Health** | **Wildfires and wildfire smoke** <https://ncceh.ca/resources/subject-guides/wildfires-and-wildfire-smoke#h2-0> | **Public health planning for wildfire smoke:** <https://ncceh.ca/resources/evidence-reviews/public-health-planning-wildfire-smoke> |  | **Health checks during extreme heat events:** <https://ncceh.ca/sites/default/files/NCCEH%20Extreme%20Heat%20Event%20-%20Health%20Checklist%20WEB_0.pdf> |
| **My health Alberta** | **Air Quality**  <https://myhealth.alberta.ca/Alberta/Pages/wildfire-smoke-health.aspx>  **Breathing Smoke or Fumes**  <https://myhealth.alberta.ca/health/pages/conditions.aspx?Hwid=sig254797> |  |  |  |
| **Alberta Health Services** | **Air Quality Events: Health information** <https://www.albertahealthservices.ca/news/air.aspx> | **2024 Wildfire Resources Information for Albertans** <https://www.albertahealthservices.ca/news/Page14070.aspx>  **How to reduce the effects of smoke-related illness** <https://www.youtube.com/watch?v=bB8zLmxvvyg>  **Wildfire smoke and your mental health** <https://www.albertahealthservices.ca/assets/info/amh/if-amh-mhpip-disaster-wildfire-smoke-mental-health-public.pdf>  **Infant Feeding in Emergencies** <https://www.albertahealthservices.ca/assets/news/advisories/ne-pha-infant-feeding-during-emergencies.pdf>  **Wildfire Smoke and Your Mental Health** <https://www.albertahealthservices.ca/assets/info/amh/if-amh-mhpip-disaster-wildfire-smoke-mental-health-public.pdf> |  |  |
| **Government of Alberta** | **Smog facts: chrome-** <https://open.alberta.ca/dataset/3e7862da-3120-4a87-aeb7-147cd469c481/resource/b71c7a69-d6fb-43ac-b68d-c0b0fccc5100/download/smogfacts-mar15-2017.pdf> |  |  |  |
| **Calgary region airshed zone** |  | **Community Guide to Wildfire Smoke and Health** <https://craz.ca/community-guide-to-wildfire-smoke-and-health/> |  |  |
| **BC Center for Disease Control** | **Wildfire Smoke** <http://www.bccdc.ca/health-info/prevention-public-health/wildfire-smoke> | **Guidance for BC Public Health Decision Makers During Wildfire Smoke Events** <http://www.bccdc.ca/resource-gallery/documents/guidelines%20and%20forms/guidelines%20and%20manuals/health-environment/wfsg_bc_guidance_2014_09_03trs.pdf#:~:text=This%20document%20provides%20a%20summary%20and%20interpretation%20of,public%20health%20decision%20making%20during%20wildfire%20smoke%20events>.  **Prepare for the worst: Learning to live with wildfire smoke** <https://nexuswebcast.mediasite.com/Mediasite/Showcase/bc-cdc-showcase/Presentation/bd7fb7d9764b41108de63428e8c421381d>  **Wildfire smoke during extreme heat events** <http://www.bccdc.ca/resource-gallery/Documents/Guidelines%20and%20Forms/Guidelines%20and%20Manuals/Health-Environment/BCCDC_WildFire_FactSheet_HotWeather.pdf>  **How to prepare for the wildfire smoke season** <http://www.bccdc.ca/resource-gallery/Documents/Guidelines%20and%20Forms/Guidelines%20and%20Manuals/Health-Environment/BCCDC_WildFire_FactSheet_HowToPrepare.pdf> |  |  |
| **The Government of British Columbia** | **Air Quality Health Index - What's the air like today?** <https://www.env.gov.bc.ca/epd/bcairquality/data/aqhi-table.html> | **BC Health and Smoke Exposure (HASE) Coordination Committee Guideline** <http://www.bccdc.ca/resource-gallery/Documents/Guidelines%20and%20Forms/Guidelines%20and%20Manuals/Health-Environment/BC%20Health%20Wildfire%20Smoke%20Response%20Coordination%20Guideline.pdf> |  |  |
| **Manitoba health** |  | **Interim Guidelines for Protecting Community Health and Wellbeing** <https://www.gov.mb.ca/health/publichealth/environmentalhealth/docs/wildlandfiresmokeexposure.pdf> |  |  |
| **Newfoundland and Labrador Department of Health and Community Services** |  | **Forest fire smoke and air quality public health guidelines** <https://www.lghealth.ca/wp-content/uploads/2018/04/Forest-Fire-Smoke-and-Air-Quality-Revised-June-2015.pdf> |  |  |
| **Northwest Territories Health and Social Services** | **Wildfire smoke exposure advisory** <https://www.hss.gov.nt.ca/en/services/environmental-health/wildfire-smoke-exposure-advisory> |  |  |  |
| **Government of Northwest Territories** |  | **Smoke Exposure from Wildfire: Guidelines for Protecting Community Health and Wellbeing** <https://www.hss.gov.nt.ca/sites/hss/files/smoke-exposure-wildfire-guidelines.pdf> |  |  |
| **Government of Ontario** | **Wildfire Smoke and Air Quality Health Reference Document** <https://www.ontario.ca/page/wildfire-smoke-and-air-quality-health-reference-document> |  |  |  |
| **Government of Quebec** | **Health Impacts of Particles from Forest Fires** <https://www.inspq.qc.ca/en/publications/1793> |  |  |  |
| **Government of Saskatchewan** | **Forest Fire Smoke and Your Health** <https://www.saskatchewan.ca/residents/environment-public-health-and-safety/environmental-health/forest-fire-smoke-and-your-health> |  |  |  |
| **Saskatchewan Health Authority** | **Protect Yourself from Summer Heat and Wildfire Smoke**  <https://www.saskhealthauthority.ca/news-events/news/protect-yourself-summer-heat-and-wildfire-smoke> |  |  |  |
| **Saskatchewan Public Safety Agency** | **Wildfire Smoke and Air Quality**  <https://www.saskpublicsafety.ca/emergencies-and-response/wildfire-smoke-air-quality> | **Guidelines for Health Staff in Northern Saskatchewan** <https://www.canada.ca/en/public-health/services/publications/healthy-living/wildfires-canada-toolkit-public-health-authorities.html> |  |  |
| **Yukon Health and Social Services** |  | **Yukon Wildfire Smoke Response Guideline**  <https://yukon.ca/sites/default/files/hss/hss-imgs/yukon_wildfire_smoke_response_guidelines_2020.pdf> |  |  |

**Other institutions**

| **Stakeholder** | **General information and Warning systems** | **Resources (e.g. fact sheets/ guidance)** | **LFS action plans** | **Other action plans** |
| --- | --- | --- | --- | --- |
| **Fire Smoke Canada** | **Fire Smoke Canada**  [**https://firesmoke.ca/**](https://firesmoke.ca/) |  |  |  |
| **Canada Red Cross** | **Breathing Emergencies: Wildfire Smoke and Poor Air Quality** [**https://www.redcross.ca/blog/2024/5/breathing-emergencies-wildfire-smoke-and-poor-air-quality**](https://www.redcross.ca/blog/2024/5/breathing-emergencies-wildfire-smoke-and-poor-air-quality) |  |  |  |
| **Sports organisation** | **UNDERSTANDING AIR QUALITY:** <https://sirc.ca/wp-content/uploads/2023/02/Air-Quality-Guiding-Document-FINAL-EN.pdf> |  |  |  |

**Supplementary Materials**

**Health management planning for landscape fire smoke events: A literature review and suggested human-centred framework for resource development**

Anna M Gjedrem^1,4^, Fay H Johnston^1,2,3,4^, Sharon L Campbell^1,2,3,4^

^1^Fire Centre, University of Tasmania, Hobart, Tasmania, Australia

^2^Menzies Institute for Medical Research, University of Tasmania, Hobart, Tasmania, Australia

^3^Centre for Safe Air, NHMRC Centre for Research Excellence, Hobart, Tasmania, Australia

^4^Healthy Environments and Lives National Research Network, University of Canberra, Canberra, ACT, Australia

D. **Resource library – LFS and health impacts in USA**

**Healthcare institutions**

| **Stakeholder** | **General information and Warning systems** | **Resources (e.g. fact sheets / courses / multimedia)** | **LFS action plans** | **Other action plans** |
| --- | --- | --- | --- | --- |
| **Healthy children** | **Wildfires: What Parents Need to Know** <https://www.healthychildren.org/English/safety-prevention/at-home/Pages/Wildfires-Information-for-Parents.aspx> |  |  |  |
| **American College of Emergency Physicians and American Academy of Paediatrics** |  | **Guidelines: Disaster Preparedness for Children and Youth with Special Health Care Needs.** https://www.aap.org/en/patient-care/disasters-and-children/professional-resources-for-disaster-preparedness/preparedness-for-children-and-youth-with-special-health-care-needs/ |  |  |
| **Family Voices** |  | **Are You Ready? Preparing for Emergencies: Tips for Families** <https://familyvoices.org/wp-content/uploads/2018/06/EmergencyDisasterPlanning_FamilyHandout.pdf> |  |  |
| **American Academy of Allergy Asthma and Immunology** | **Wildfire Smoke, Asthma and the Immune System**  <https://www.aaaai.org/tools-for-the-public/conditions-library/asthma/wildfire> |  |  |  |
| **American Lung Association** | **Wildfires**  <https://www.lung.org/clean-air/emergencies-and-natural-disasters/wildfires> | **Protect your health during wildfires:** <https://www.lung.org/getmedia/695663e2-bdb8-4a61-9322-02657f530b99/Protect-Your-Health-During-Wildfires-5-29-2020-(1).pdf> |  | **Create an Asthma Action Plan:** <https://www.lung.org/lung-health-diseases/lung-disease-lookup/asthma/managing-asthma/create-an-asthma-action-plan>  **COPD Action and management plan:** <https://www.lung.org/lung-health-diseases/lung-disease-lookup/copd/living-with-copd/copd-management-tools> |
| **Boston Children’s Hospital Paediatric Environmental Health Centre** |  |  |  | **Action plan: Your health, climate change, and extreme weather:** [www.childrenshospital.org/programs/pediatric-environmental-health-center/climate-rx/extreme-weather](http://www.childrenshospital.org/programs/pediatric-environmental-health-center/climate-rx/extreme-weather) |
| **noattacks** |  |  |  | **Asthma Action Plan**  <https://www.noattacks.org/asthma-action-plan> |
| **National Environmental Education Foundation (NEEF)** |  | **Course: Environmental Management of Pediatric Asthma: Guidelines for Healthcare Providers.** <https://neef.inquisiqlms.com/catalog/>  **How Wildfire Smoke Affects Asthma and What You Can Do to Stay Safe**  <https://www.neefusa.org/story/health-and-environment/how-wildfire-smoke-affects-asthma-and-what-you-can-do-stay-safe> |  |  |

**Research institutions**

| **Stakeholder** | **General information and Warning systems** | **Resources (e.g. fact sheets / courses / multimedia)** | **LFS action plans** | **Other action plans** |
| --- | --- | --- | --- | --- |
| **University of California, San Francisco. PEHSU Pediatric Environmental Health Speciality Units** | **Information. Guidelines and infographics:** <https://www.pehsu.net/health_professionals_families/health_topics/wildfires/> | **PEHSU Information on Health Risks of Wildfires for Children – Acute Phase Guidance for Health Professionals:** [**https://wspehsu.ucsf.edu/wp-content/uploads/2015/10/wildfire_acute_2011_health_prof.pdf**](https://wspehsu.ucsf.edu/wp-content/uploads/2015/10/wildfire_acute_2011_health_prof.pdf)  **Wildfire smoke affects children’s health. Here’s 3 tips**  [**https://wspehsu.ucsf.edu/wp-content/uploads/2021/08/wfire_sitebanner_1200x628_logo.jpg**](https://wspehsu.ucsf.edu/wp-content/uploads/2021/08/wfire_sitebanner_1200x628_logo.jpg)  How to reduce wildfire smoke exposure for kids and pregnant people  <https://wspehsu.ucsf.edu/wp-content/uploads/2020/08/wildfireinfographic081920.pdf>  WILDFIRE SMOKE FACTSHEET Children’s Health and Wildfires A Resource for Families  <https://www.pehsu.net/lib_facts/WILDFIRE_SMOKE_FACTSHEET-113023CLEAN_LOGO.pdf>  Protecting Kids from Wildfire Smoke: Actions for California Schools  <https://wspehsu.ucsf.edu/wp-content/uploads/2021/11/wildfire-action-for-ca-schools_stanford.pdf> |  |  |

**Government authorities**

| **Stakeholder** | **General information and Warning systems** | **Resources (e.g. fact sheets/ guidance)** | **LFS action plans** | **Other action plans** |
| --- | --- | --- | --- | --- |
| **U.S. Environmental Protection Agency** | **EPA's Smoke Sense:** <https://www.epa.gov/air-quality/wildland-fires-and-smoke> | **Smoke-Ready Communities:** <https://www.epa.gov/sciencematters/partners-collaborate-smoke-ready-communities-research-enhance-local-readiness>  **Smoke-Ready Toolbox for Wildfires:** <https://www.epa.gov/air-research/smoke-ready-toolbox-wildfires>  **Course Wildfire smoke and your patients’ health:** <https://www.epa.gov/wildfire-smoke-course>  **Course: Particle Pollution and Your Patients' Health:** <https://www.epa.gov/pmcourse>  **Course: Ozone and Your Patients' Health**  <https://www.epa.gov/ozone-pollution-and-your-patients-health>  **Video: Wildfire Smoke: A Guide for Public Health Officials** <https://www.youtube.com/watch?v=waMXCFMqYnA>  **Heart Disease, Stroke, and Outdoor Air Pollution**  <https://www.airnow.gov/sites/default/files/2018-03/heartflyer.pdf>  **Asthma and outdoor air pollution**  <https://www.airnow.gov/sites/default/files/2018-03/asthma-flyer.pdf>  **Children’s book: Why is Coco Red?** <https://document.airnow.gov/why-is-coco-red.pdf> |  |  |
| **U.S. Centre for Disease Control and Prevention** | **Wildfires and Your Safety:** <https://www.cdc.gov/wildfires/about/index.html>  **About air quality:** <https://www.cdc.gov/air-quality/about/index.html>  **How Wildfire Smoke Affects Your Body:** <https://www.cdc.gov/wildfires/risk-factors/index.html>  **Wildfire Smoke and People with Chronic Conditions:** <https://www.cdc.gov/wildfires/risk-factors/wildfire-smoke-and-people-with-chronic-conditions.html> | **Safety Guidelines: Wildfires and Wildfire Smoke:** <https://www.cdc.gov/wildfires/safety/how-to-safely-stay-safe-during-a-wildfire.html>  **Personal Needs:** <https://www.cdc.gov/prepare-your-health/take-action/personal-needs.html>  **Wildfire Smoke and Pregnancy**  <https://www.cdc.gov/wildfires/risk-factors/wildfire-smoke-and-pregnancy.html>  [Español](https://www.cdc.gov/wildfires/es/risk-factors/humo-de-los-incendios-forestales-y-los-ninos.html)  **Wildfire Smoke and Children**  <https://www.cdc.gov/wildfires/risk-factors/wildfire-smoke-and-children.html>  **Wildfire Smoke and People with Chronic Conditions**  <https://www.cdc.gov/wildfires/risk-factors/wildfire-smoke-and-people-with-chronic-conditions.html> |  |  |
| **U.S. Fire Administration Working for fire-safe America** | **Minimising the Effects of Wildfire Smoke**  <https://www.usfa.fema.gov/blog/minimizing-the-effects-of-wildfire-smoke/>  **Warnings and monitoring: air quality tools:** <https://portal.airfire.org/> | **Interagency Wildland Fire Air Quality Response Program:** <https://www.wildlandfiresmoke.net/home>  **Smoke ready brochure** <https://drive.google.com/file/d/1znBiiRmNRdVHJCBwQIc0p7ZNQ7pQqwgR/view> |  |  |
| **AirNow** | **Wildfire Guide Information:** <https://www.drought.gov/data-maps-tools/airnow-fire-and-smoke-map> | **Smoke advisories:** <https://www.airnow.gov/air-quality-and-health/fires/smoke-advisories/>  **Video: Protecting Children from Wildfire Smoke in the Pacific Southwest** <https://www.youtube.com/watch?v=yjjDSDfonik>  **Wildfire guide fact sheets:**  <https://www.airnow.gov/wildfire-guide-factsheets/#prepare-for-fire-season>  **Wildfire Smoke: A Guide for Public Health Officials:** <https://www.airnow.gov/publications/wildfire-smoke-guide/wildfire-smoke-a-guide-for-public-health-officials/>  **Online training for health professionals:** <https://www.airnow.gov/air-quality-and-health/online-training-for-health-professionals/> |  |  |
| **National Heart, Lung and Blood Institute** |  |  |  | **Asthma action plan:** [www.epa.gov/system/files/documents/2023-04/Asthma-Action-Plan-2020_rev_508.pdf](http://www.epa.gov/system/files/documents/2023-04/Asthma-Action-Plan-2020_rev_508.pdf) |
| **Airknowledge** |  | **Course: Resources to Aid in Protecting Human Health from Wildland Fire Smoke:** <https://airknowledge.gov/Mod/Resources_to_Aid_in_Protecting_Human_Health_from_Wildland_Fire_Smoke/Web/index.html#/lessons/-1kOOvOdCRxhSp1WCnMx2ZgFEkQnX4jD> |  |  |
| **Particulate Pollution Health Advisory Program** | **Wildfire Smoke Recommendations**  [https://www.gbuapcd.org/AirMonitoringData/Smoke/smokyConditionsRecs.html#](https://www.gbuapcd.org/AirMonitoringData/Smoke/smokyConditionsRecs.html) |  |  |  |
| **U.S. Forest Service** | **National Integrated Drought Information System**  <https://www.drought.gov/about/partners/us-forest-service> |  |  |  |
| **United States Department of Agriculture** |  | **Smoke Ready**  <https://wildfirerisk.org/reduce-risk/smoke-ready/>  **Wildfire smoke and COVID-19: Frequently asked questions**  <https://www.nifc.gov/sites/default/files/pio/documents/Smoke_COVID19_FS_FactSheet.pdf> |  |  |
| **Ready National public service campaign** |  | **Plan for wildfires:** <https://www.ready.gov/wildfires> |  |  |
| **Alaska Division of Air Quality, Department of Environmental Conservation** | **Air quality index**  <https://experience.arcgis.com/experience/8655256b056a48f6a19788d43cdab30f>  **Wildfire Smoke Information**  <https://dec.alaska.gov/air/anpms/wildfire-smoke/> |  |  |  |
| **AK Dept. of Health & Social Services** | **Wildfires and Your Health**  <https://health.alaska.gov/pages/wildfires.aspx> |  |  |  |
| **California Air Resources Board** | **Southern California Wildfire Response**  <https://ww2.arb.ca.gov/> | **Smoke ready California** <https://ww2.arb.ca.gov/smokereadyca> |  |  |
| **California Department of Public Health** | **Smoke & Ash Safety** <https://www.cdph.ca.gov/Programs/EPO/Pages/Wildfire%20Pages/Smoke-Ash-Safety.aspx> | **Wildfire Smoke Considerations for California’s Public Health Officials August 2022:** <https://www.cdph.ca.gov/Programs/EPO/CDPH%20Document%20Library/EOM%20Documents/Wildfire-Smoke-Considerations-CA-PHO_08-2022.pdf> |  |  |
| **Listos California** |  | **Resource hub:** <https://www.listoscalifornia.org/resources/?_search=disaster%20ready%20guide> |  |  |
| **State of California OEHHA** | **Wildfires:** <https://www.oehha.ca.gov/public-information/general-info/wildfires> |  |  |  |
| **CalEPA California Environmental Protection Agency** | **Fire Response and Recovery**  <https://calepa.ca.gov/disaster/fire/> |  |  |  |
| **Idaho Department of Environmental Quality** | **Real-Time Air Monitoring** <https://airquality.deq.idaho.gov/home/map> |  |  |  |
| **ID Dept. of Health & Welfare** | **Wildfire Smoke**  <https://healthandwelfare.idaho.gov/health-wellness/environmental-health/wildfire-smoke> |  |  |  |
| **Illinois Department of Public Health** | **Wildfire Smoke**  <https://dph.illinois.gov/topics-services/environmental-health-protection/wildfire-smoke.html> |  |  |  |
| **Montana Department of Environmental Quality** | **MONTANA WILDFIRE SMOKE**  [**https://www.montanawildfiresmoke.org/**](https://www.montanawildfiresmoke.org/) |  |  |  |
| **New Mexico Fire Information** | **Smoke management**  <https://nmfireinfo.com/smoke-management/> |  |  |  |
| **New York State Department of Health** | **Exposure to Smoke from Fires**  <https://www.health.ny.gov/environmental/outdoors/air/smoke_from_fire.htm> |  |  |  |
| **NY Pediatric Environmental Health Specialty Unit** |  | **What to do if wildfire smoke is affecting your area:** <https://nyscheck.org/wp-content/uploads/2023/06/WildfireSmoke_2023.pdf>  **Guidelines for careers of young children: Outdoor Air Quality – Wildfire Smoke** <https://nyscheck.org/outdoor-air-quality-wildfire-smoke/> | **New York State Prescriptions for Prevention - Outdoor air quality action plan**  <https://nyscheck.org/rx/> |  |
| **Oregon Health Authority** | **Wildfires and Smoke**  <https://www.oregon.gov/oha/ph/preparedness/prepare/pages/prepareforwildfire.aspx> | **Wildfire Smoke and Your Health** <https://www.oregon.gov/oha/ph/Preparedness/Prepare/Documents/OHA%208626%20Wildfire%20FAQs-v6c.pdf> |  |  |
| **Oregon Rulemaking** |  | **Wildfire smoke – partner toolkit:** <https://doh.wa.gov/community-and-environment/air-quality/smoke-fires/partner-toolkit> |  |  |
| **Oregon Department of Environmental Quality** | **OR Air Quality Monitoring Map** <https://aqi.oregon.gov/> |  |  |  |
| **The State of Washington, Department of Health** |  | **Washington Air Quality Guide for Particle Pollution:** <https://doh.wa.gov/sites/default/files/2024-06/821-174.pdf>  **Recommendations for wildfire smoke and respiratory viruses:** <https://doh.wa.gov/sites/default/files/legacy/Documents/Pubs/821-144.pdf?uid=64c14163ebe3d>  **Washington guide for public health actions for wildfire smoke:** <https://doh.wa.gov/sites/default/files/legacy/Documents/4300/334-429-WAQAGuidePublicHealthActions.pdf?uid=6278e859bc4b4>  **What should I Expect from my health care provider?** <https://doh.wa.gov/you-and-your-family/illness-and-disease-z/asthma/what-should-i-expect-my-health-care-provider>  **Wildfire Smoke - Partner Toolkit**  <https://doh.wa.gov/community-and-environment/air-quality/smoke-fires/partner-toolkit> |  |  |
| **Washington Department of Ecology** | **Washington air quality map** <https://enviwa.ecology.wa.gov/mobile/> |  |  |  |
| **Washington Department of Natural Resources** | **Wildfire Smoke**  <https://www.dnr.wa.gov/smoke> | **Community Wildfire Resilience Resource Library** <https://www.dnr.wa.gov/community-wildfire-resilience-resource-library> |  |  |

**Other institutions**

| **Stakeholder** | **General information and Warning systems** | **Resources (e.g. fact sheets / courses / multimedia)** | **LFS action plans** | **Other action plans** |
| --- | --- | --- | --- | --- |
| **American Red Cross** |  | **Learn how to keep your family and home safe during a wildfire:** <https://www.redcross.org/get-help/how-to-prepare-for-emergencies/types-of-emergencies/wildfire.html>  **Wildfire Preparedness Checklist:** <https://www.redcross.org/content/dam/redcross/get-help/pdfs/wildfire/EN_Wildfire-Safety-Checklist.pdf> |  |  |
